# Sex differences in human umbilical vein endothelial cells following ox-LDL injury

**DOI:** 10.1186/s13293-026-00845-5

**Published:** 2026-02-07

**Authors:** Camilla Cittadini, Elisabetta Straface, Ilaria Campesi, Lucrezia Gambardella, Giampiero Capobianco, Letizia Barbieri, Laura Doro, Flavia Franconi, Giulio Testone, Rosa Vona

**Affiliations:** 1 Center for Gender-Specific Medicine, Italian National Institute of Health, Viale Regina Elena, Rome, 299-00161 Italy; 2 Department of Biomedical Sciences, University of Sassari, Viale San Pietro 43, Sassari, 07100 Italy; 3 Laboratory of Gender Pharmacology and Medicine, National Institute of Biostructures and Biosystems, Sassari, 07100 Italy; 4 Department of Medicine, Surgery and Pharmacy, University of Sassari, Viale San Pietro 43, Sassari, 07100 Italy; 5 Gynecologic and Obstetric Clinic, AOU, Viale San Pietro 12, Sassari, 07100 Italy; 6 Institute for Biological Systems, National Research Council (CNR), Via Salaria Km 29,300, Monterotondo, Rome, 00015 Italy

**Keywords:** Cardiovascular diseases, Atherosclerosis, Sex differences, Oxidized low-density lipoproteins (ox-LDL), Male human umbilical vein endothelial cells (MHUVECs), Female human umbilical vein endothelial cells (FHUVECs), Endothelial dysfunction

## Abstract

**Background:**

Numerous sex differences has been described in aterosclerosis including in endothelial dysfunction. Oxidized low-density lipoproteins (ox-LDL) contribute to the formation of atherosclerotic plaque by binding to a membrane glycoprotein expressed by endothelial cells. Ox-LDL also play a key role in mediating endothelial dysfunction during pregnancy. Elevated maternal ox-LDL levels can lead to oxidative stress, inflammation and apoptosis in placental and fetal endothelial cells. The aim of this study was to investigate sex-related differences in the response to ox-LDL-induced damage *in human umbilical vein endothelial cells (HUVECs) isolated from male and female newborns.*

**Methods:**

In our study, the effects of *100 µg/ml* ox-LDL on HUVECs, obtained from umbilical cords of healthy newborns of both sexes, were analyzed. By flow cytometry, fluorescence microscopy, and Western blotting techniques, mitochondrial function, cell survival, and autophagy were studied.

**Results:**

Sex differences in cell motility and fate have been detected after ox-LDL treatment. Indeed, following ox-LDL treatment, *male HUVECs (MHUVECs*) exhibited reduced motility and a significant increase in adhesion molecules ICAM-1 and VCAM-1, in contrast to *female HUVECs (FHUVECs).* Furthermore, MHUVECs exhibited higher levels of fission proteins (DRP1 and Fis1), superoxide anion (O₂⁻), and earlier mitochondrial membrane (MM) hyperpolarization, while FHUVECs showed higher levels of fusion proteins (OPA1 and MFN2), hydrogen peroxide (H₂O₂), and delayed MM changes. These findings were consistent with a greater propensity for apoptosis in MHUVECs. In contrast, FHUVECs exhibited higher levels of Survivin, making them less susceptible to apoptosis and more susceptible to the autophagy process.

**Conclusions:**

Our findings reveal significant sex-related variations in endothelial responses to oxidative stress. The enhanced survival and repair capacity of FHUVECs suggests that female cells are more resilient to ox-LDL-induced damage.

**Supplementary Information:**

The online version contains supplementary material available at 10.1186/s13293-026-00845-5.

## Abstract (plain language summary)

Atherosclerosis, a major cause of cardiovascular disease, is a condition in which arteries become obstructed by fatty deposits, which often begin with damage to endothelial cells that line blood vessels. One of the main causes of this condition is oxidized low-density lipoproteins (ox-LDL), harmful form of cholesterol that triggers inflammation, oxidative stress and cell death. These processes can contribute to the development and progression of atherosclerotic plaque. This study investigated the effects of ox-LDL on *human umbilical vein endothelial cells (HUVECs)* from healthy male and female newborns. The data showed that ox-LDL reduce cell movement and increase oxidative stress in both sexes, but male cells were more vulnerable than female cells. Male cells exhibited higher levels of reactive oxygen species, exhibited earlier and more severe mitochondrial damage, and underwent greater apoptotic cell death. They also exhibited higher levels of proteins associated with inflammation and impaired cell repair. In contrast, female cells were better at adapting to the stress caused by ox-LDL. They maintained healthier mitochondria, exhibited stronger repair responses and activated protective processes such as autophagy, which helps to remove damaged components. These sex-related differences suggest that male and female fetuses may respond differently to cholesterol-related damage even before birth. This could influence their future risk of cardiovascular disease. Understanding these differences could inform the development of more personalized approaches to preventing heart disease from early life onwards. This could also provide potential biomarkers and/or innovative preventive approaches to combat the onset of cardiovascular disease from the womb onwards, ensuring personalized care.

**Supplementary Information:**

The online version contains supplementary material available at 10.1186/s13293-026-00845-5.

## Highlights

- This study examined *how male and female human umbilical vein endothelial cells (MHUVECs*,* FHUVECs)* responded differently to ox-LDL-induced damage.
- Ox-LDL reduced cell motility and increased oxidative stress and apoptosis, particularly in male cells.
- MHUVECs showed higher levels of inflammatory markers (ICAM-1, VCAM-1), mitochondrial damage, and cell death pathways (Caspase-3, Bax).
- FHUVECs exhibited better repair capacity, increased mitochondrial fusion, and higher Survivin expression, which protects against apoptosis.
- Reactive oxygen species production and mitochondrial membrane changes occurred earlier and more severely in male cells.
- Only male cells showed impaired autophagy, with increased p62 accumulation, while female cells maintained functional autophagy.
- The data obtained suggest sex-dependent cellular responses to cholesterol-induced stress begin in fetal life and may influence future cardiovascular risk.

**Supplementary Information:**

The online version contains supplementary material available at 10.1186/s13293-026-00845-5.

## Introduction

Endothelial dysfunction is a key event in the initiation and progression of atherosclerosis, a chronic arterial disorder caused by lipid accumulation and plaque formation within the vessel wall [1]. Plaque growth narrows the arterial lumen, restricts blood flow, and may lead to thrombus formation upon rupture, resulting in myocardial infarction or stroke.

Atherosclerotic lesions are rich in oxidized low-density lipoproteins (ox-LDL), generated by oxidative modification of LDL particles within the intima. Ox-LDL bind to the lectin-like oxidized LDL receptor-1 (LOX-1) on endothelial cells (ECs), activating oxidative and inflammatory pathways that induce apoptosis, foam cell formation, and loss of endothelial integrity [2–4].

Ox-LDL play a key role in mediating endothelial dysfunction during pregnancy. Elevated maternal ox-LDL promote oxidative stress, inflammation, and apoptosis in placental and fetal ECs, involving activation of mitochondrial and inflammatory pathways [2, 3]. These processes impair nitric oxide bioavailability, increase vascular permeability, and alter angiogenic signaling, thereby contributing to adverse pregnancy outcomes such as preeclampsia and intrauterine growth restriction [5, 6]. Moreover, in utero exposure to ox-LDL contributes to early vascular programming and pro-atherogenic remodeling in the fetal endothelium, potentially predisposing offspring to long-term cardiovascular risk [7, 8]. In addition, Ox-LDL exert potent oxidizing effects, increasing reactive oxygen species (ROS) generation in cultured endothelial cells (ECs) [9].

Mitochondria are the main cellular source of ROS in cells. Their dysfunction in ECs, vascular smooth muscle cells, and macrophages can induce high levels of oxidative stress, increased production of ROS and cell apoptosis that contribute to the formation of advanced atherosclerotic plaque [10]. Mitochondria are highly dynamic structures and undergo morphological changes and spatial rearrangements to maintain cellular homeostasis through coordinated cycles of mitochondrial fusion and fission, commonly referred to as mitochondrial dynamics. They control the number, size, shape, and distribution of mitochondria within the cells [11]. Mitochondrial fusion consists in the fusion of two originally distinct mitochondria into a single organelle, whereas mitochondrial fission is the division of a single defective mitochondrion into two or more individual mitochondrial units. Fusion alleviates stress by mixing the contents of partially damaged mitochondria, while fission is required to create new mitochondria, remove damaged ones, and facilitate apoptosis during high levels of cellular stress [12]. The first is a process that requires three key proteins: mitofusin 1 and 2 (MFN1 and MFN2) and optic atrophy 1 (OPA1); the second is a process promoted by the dynamin-related-like protein 1 (DRP1). Damaged and depolarized mitochondria are eliminated by mitophagy, a selective form of autophagy involving the degradation and recycling of cellular components, small organelles and damaged proteins to provide essential nutrients for cell survival [13]. However, excessive or defective activity of this physiological process is not compatible with cellular homeostasis [12]. Indeed, aberrant autophagy is generally associated with a wide range of pathologies, including cardiovascular disease [14–16]. Impaired mitophagy leads to a surplus of defective organelles within cells and increased release of ROS, which contributes to impaired normal mitochondrial respiration, along with cell death. It has been demonstrated that in human umbilical vein endothelial cells (HUVECs) ox-LDL induce mitochondrial DNA damage resulting in HUVECs apoptosis [17].

Sex differences in the incidence, progression, and complications of atherosclerosis have been well documented in adults over the past decade [18–21], and evidence from histological studies indicates that the formation of atherosclerotic lesions, such as fatty streaks, can begin during fetal development [7, 8]. However, the mechanisms underlying sex-dependent differences in prenatal endothelial responses remain poorly understood. Male and female fetuses develop in distinct hormonal and molecular environments, and sex-chromosome–driven gene regulation shapes sex-biased developmental trajectories from early gestation [22]. Multiomic studies show widespread sex influence on gene expression across placental trophoblasts, immune cells, and ECs, with females favoring immune and growth pathways and males prioritizing nutrient transport [20, 23]. These findings highlight a fundamental sex dimorphism in utero that may contribute to lifelong differences in vascular and metabolic health [24–27].

Moreover, HUVECs are directly exposed in utero to oxidative stressors such as ox-LDL, which contribute to endothelial dysfunction in pregnancy complications and to early vascular programming [2, 28, 29].

Accordingly, this study aims to investigate sex differences in oxidative damage induced by ox-LDL in HUVECs derived from male and female umbilical cords.

HUVECs are widely used to study in vitro endothelial damage and sex differences in the development of cardiovascular disease.

Literature data report that HUVECs obtained from male and female umbilical cords when studied independently are sexually dimorphic in their morphological, proliferative, and migratory properties [30], and differently respond to different stimuli associated with endothelial disfunction with impaired migration, autophagy and mitophagy [31–35].

## Materials and methods

### Donors

Umbilical cords were collected from healthy term (37–42 weeks) male (*n* = 3) and female (*n* = 3) newborns delivered vaginally at the Obstetrics and Gynaecology Clinic, University of Sassari. Mothers were healthy, non-obese, non-smoking, and drug-free except for folic acid and iron supplementation. Only cords from neonates with normal birth weight (2,430–4,050 g for females; 2,550–4,190 g for males, 10th–90th percentiles according to INeS charts [36] were used for HUVECs isolation. Informed consent was obtained from all mothers in accordance with the Declaration of Helsinki.

Umbilical cord collection was approved by the Independent Ethical Committee of Azienda Ospedaliero Universitaria (AOU Cagliari; prot. PG/2018/18480).

### Cell isolation, characterization and treatments

Primary female HUVECs (FHUVECs) and male HUVECs (MHUVECs) were isolated by collagenase treatment (Sigma-Aldrich, Milano, Italy), as previously described [30], and cultured in plates pre-coated with 1% gelatine (Sigma-Aldrich, Milano, Italy) in M199 medium (Life Technologies, Monza, Italy) supplemented with 10% fetal bovine serum (FBS) (Life Technologies, Monza, Italy), 10% newborn calf serum (NBCS) (Life Technologies, Monza, Italy), 1% antibiotic/antimicotic (Sigma-Aldrich, Milano, Italy) and 2 mM of L-glutamine (Sigma-Aldrich, Milano, Italy) in a 5% CO2 humidified atmosphere. As previously described, cultured cells were characterized as ECs by the exhibition of cobblestone morphology when they were contact- inhibited and by an evaluation of the expression of von Willebrand factor, a glycoprotein that is constitutively stored in intra-endothelial Weibel-Palade granules [30].

FHUVECs and MHUVECs were used from passages 3–5 to ensure their endothelial characteristics, and all experiments were conducted in duplicate or triplicate.

Male and female cells were treated with 25, 50, 100, and 200 µg/ml of ox-LDL (Thermo Fisher Scientific, Ma, USA) to study cell viability (Supplementary Fig. 1).

For the study we selected ox-LDL at concentration of 100 µg/ml, concentration used in the literature to evaluate endothelial dysfunction [37].

Cells were treated for 6 and 12 h to evaluate oxidative stress or 24 h to evaluate functional changes.

The samples were randomized prior to experimental processing. Specifically, the order of the samples was randomized using a random number generator before analysis. Data analysis was performed blind with respect to sample identity and experimental group until all primary analyses were complete.

### Cell viability assay

Cell viability was assessed using the MTT assay, in which MTT reagent (0.5 mg/ml) was added and incubated for 4 h at 37 °C. The resulting formazan crystals were dissolved in DMSO, and absorbance was measured at 570 nm using a microplate reader. Cell viability was calculated relative to the untreated control group. All treatments were performed in triplicate, and data represent the mean ± SD from at least three independent experiments.

### Cell migration

Cell migration was examined with a scratch assay, according to Liang et al. [38], and performed as described [39]. Approximately 2.5 × 10^5^ cells were seeded into a 24-well plate. When the cells reached confluence were scratched with a sterile 20 µl pipette tip. The cells were then washed with PBS and photographed at T0. The migration of cells toward the wound closure of the same region after 24 h was monitored, and images (10 fields/dish) were acquired using a digital camera system coupled with an inverted microscope (IX-71 Olympus Corporation, Tokyo, Japan). Repopulation by migrating cells of the wound region was then analyzed and quantified using the ImageJ v1.48 software.

### Flow cytometry

#### Adhesion molecules

The levels of ICAM-1 and VCAM-1 proteins were analyzed using flow cytometry. After 24 h of ox-LDL treatment, the cells were fixed with 4% paraformaldehyde for at least 2 h and then permeabilized with 0.5% Triton X-100 (Sigma Aldrich, St. Louis, Missouri, USA) in PBS for 5 min. After washing in PBS, the cells were stained with anti-ICAM-1 or anti-VCAM-1 primary antibodies (all Santa Cruz Biotechnology, CA, USA, dilution 1:50) for 45 min at room temperature, followed by anti-mouse Alexa Fluor 488 (Invitrogen, dilution 1:200) for a further 30 min at 37 °C. After washing, samples were immediately analyzed on a FACScalibur cytometer (BD Biosciences) equipped with a 488 argon and with a 635 red diode laser. Data were recorded and statistically analyzed by a Macintosh computer using CellQuest software (BD Biosciences, NJ, USA).

#### Redox state evaluation

For a qualitative analysis of oxidative stress, we quantified intracellular ROS production content. The following cell-permeable fluorophores were used to stain living cells, all of which were diluted in PBS: (all from Thermo Fisher Scientific, Ma, USA): dihydroethidium (DHE, 1 µM DHE, dilution 1:500, for 15 min at 37 °C protected from light) to detect superoxide anion levels by red fluorescence; dihydrorhodamine 123 (DHR123, 10 µM, dilution 1:300, for 15 min at 37 °C protected from light) to detect hydrogen peroxide by green fluorescence; MitoSOX (5 µM, dilution 1:500, for 30 min at 37 °C protected from light) to detect mitochondrial ROS by red fluorescence.

#### Mitochondrial membrane potential (MMP)

The MMP of control and treated cells was analyzed by using 10 µM of JC-1 (5–5¢,6–6¢-tetrachloro-1,1¢,3,3¢-tetraethylbenzimidazol-carbocyanine iodide, Molecular Probes, Thermo Fisher, dilution 1:500) for 15 min, protected from light, at 37 °C in PBS, as previously described [40].

#### Evaluation of apoptotic cells

Quantitative evaluation of apoptotic cells was performed by flow cytometry after double staining with FITC- conjugated Annexin V/propidium iodide (PI) by using apoptosis detection kit (Marine Biological Laboratory, Woods Hole, MA, USA), according to the manufacturer’s instructions. The test allows to discriminate early apoptosis (AV/PI negative) from late apoptosis (double positive AV/PI) or necrosis (single PI positive).

The fluorescence signals were normalised to the number of cells. At least 20,000 events were acquired for each sample and all measurements were performed at least in triplicate as technical replicates for each biological replicate.

### Immunofluorescence microscopy

For immunofluorescence analyses, control and treated cells were fixed with 4% paraformaldehyde PBS for a least 2 h at room temperature. After washing in the same buffer, cells were permeabilized with 0.5% Triton X-100 (Sigma-Aldrich) in PBS for 5 min. Then the cells were incubated for 45 min, at room temperature, with the following antibodies: LC3 (Novus Biologicals, CO, USA; dilution 1:100), p62 (Sigma-Aldrich, dilution 1:100). As secondary antibodies AlexaFluor 488-conjugated anti-mouse IgG and AlexaFluor 594-conjugated anti-mouse IgG (both Invitrogen, Thermo Fisher, dilution 1:200) were used. To stain mitochondria, cells were incubated with 1 µM Mitotracker Red CMXRos (Invitrogen, Thermo Fisher; dilution 1:1000), 30 min before fixation with paraformaldehyde. For analysis of actin polymerization state, we used phalloidin FITC (Sigma-Aldrich, dilution 1:1000).

After washings with PBS cells were counterstained with Hoechst 33,258 (Sigma-Aldrich) to highlight the nuclei and finally mounted in fluorescence mounting medium (Dako, Glostrup, Denmark). Images were acquired with intensified video microscopy (IVM) with an Olympus fluorescence microscope (Olympus Corporation, Milan, Italy) equipped with a CoolLed pE-300-W (CoolLED Ltd., Andover, United Kingdom).

For stress fibers quantification, we conducted semi-automated image analysis using ImageJ/FIJI software. We quantified the corrected total cell fluorescence (CTCF). For each cell, a cell ROI was defined and the background was subtracted using fixed parameters. Measurements were performed on 12 cells per condition from three independent experiments.

Quantitative analysis of mitochondrial morphology was performed using the MiNA plugin in Fiji/ImageJ. Metrics including mitochondrial footprint, mean branch length and number of branches per mitochondrion were quantified. Images were acquired at 63× magnification with identical settings for acquisition, and a minimum of 16 cells per condition from three independent experiments were analyzed. Image files were randomized and analyzed in a blinded manner.

### Western blot analysis

Cells were lysed in RIPA lysis buffer, with added protease and phosphatase inhibitors. The amount of protein loaded was determined using Varioskan Lux (Thermo Scientific). 30 µg proteins were subsequently loaded onto a 10–12% polyacrylamide gel (Invitrogen, Thermo Fisher), electrotransferred onto PVDF membranes (BioRad Laboratories, CA, USA) and incubated overnight at 4 °C with the following antibodies: Bax and Survivin (all from Santa Cruz, CA, USA; dilution 1:500), OPA1, DRP1, MFN2 and Caspase-3 (all from Cell Signaling Technology, Ma, USA; dilution 1:1000), Fis1 (Enzo Life Sciences, NY, USA; dilution 1:1000), LC3 (Novus Biologicals, CO, USA, dilution 1:1000), p62 (Sigma-Aldrich, dilution 1:1000). To quantify variation in antioxidant enzymes, we used an oxidative stress defence Western blot cocktail (Abcam, dilution 1:1000) containing catalase (CAT), superoxide dismutase 1 (SOD1), thyreodoxine (Trx) and smooth muscle actin. After membranes washing, immune complexes were detected with horseradish peroxidase-conjugated species-specific secondary antibodies (Jackson Laboratory, ME, USA, dilution 1:10,000 for mouse antibodies and 1:20,000 for rabbit antibodies). Membranes were developed using ECL detection reagents (Millipore Corporation, Billerica, MA, USA). Reactive bands were detected with the ChemiDocMP system (Bio-Rad). To ensure the presence of equal amounts of protein, membranes were again probed with anti-GAPDH or anti Tubulin (all Santa Cruz). Protein expression levels were quantified using densitometric analysis with ImageJ.

### Statistical analysis

Two-way ANOVA was performed in R to evaluate main effects of treatment (T), sex (S), and their interaction (T×S) for each parameter using the aov function in R. Normality and homogeneity of variances were assessed via Shapiro-Wilk tests on residuals and Levene’s test, respectively. P-values were adjusted for multiple comparisons using the Benjamini-Hochberg FDR procedure. Significant effects (*p* ≤ 0.05) were followed by Tukey’s HSD post-hoc test, with results summarized using compact letter display where groups sharing letters do not differ significantly. All data reported in this paper were verified in at least three independent experiments and are presented as the mean ± standard deviation (SD). For flow cytometry experiments, at least 20,000 events were acquired for each sample.

## Results

### Baseline characteristics of enrolled mothers

Table 1 shows the baseline characteristics of mothers and newborns stratified by sex. Mothers of males (*n* = 3) and females (*n* = 3) newborns did not differ significantly in body weight and BMI at the beginning or end of pregnancy as well as in gestational age. No significant differences were also detected in the weight of male and female newborns.

**Table 1:** Baseline characteristics of mothers and newborns

Age of mothers (years)	Male neonates (*n* = 3)	Female neonates (*n* = 3)	*p*-value
	35.0 ± 7.8	38.7 ± 2.0	0.413
Body weight of mothers (start) (kg)	60.2 ± 5.7	54.25 ± 5.9	0.170
Body weight of mothers (end) (kg)	71.0 ± 9.7	66.2 ± 6.3	0.427
Body increase during pregnancy (Kg)	10.8 ± 5.9	12.0 ± 2.2	0.717
Body mass index (start) (Kg/m^2^)	25.4 ± 2.63	19.8 ± 1.9	0.078
Body mass index (end) (Kg/m^2^)	29.9 ± 3.1	24.2 ± 2.2	0.113
BMI increase during pregnancy (Kg/m^2^)	4.5 ± 2.5	4.4 ± 0.9	0.913
Gestational age (weeks)	40.0 ± 1.7	40.0 ± 3.0	1.00
Weight of newborns (kg)	3.0 ± 0.4	3.2 ± 0.1	0.709

Data are reported as mean ± SD

### Ox-LDL influence cell migration by altering the organization of actin and upregulating adhesion molecules in FHUVECs

**Fig. 1:**
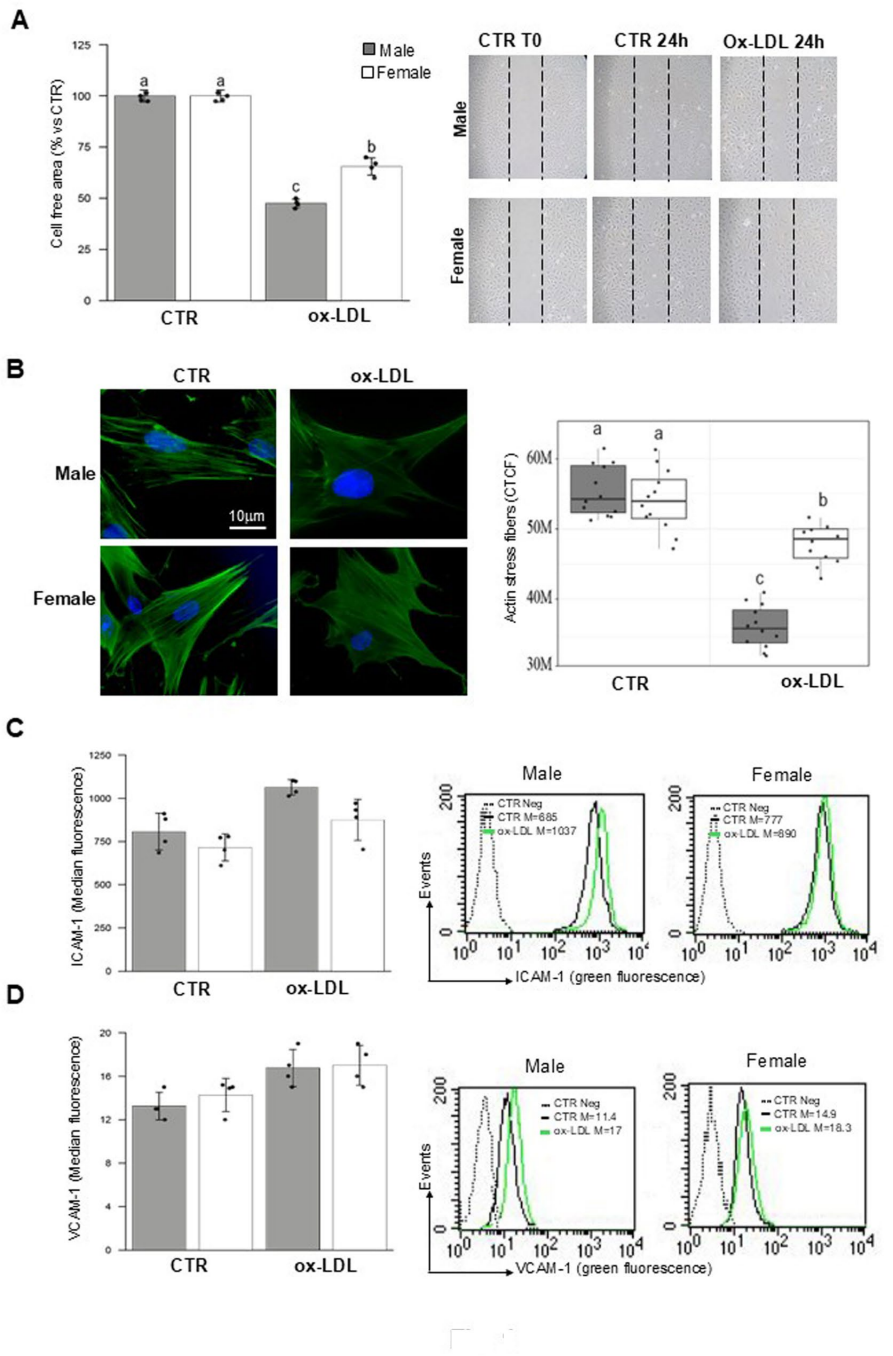
Ox-LDLs influence migration ability. (A, left panel) Migration test performed by scratch assay on FHUVECs and MHUVECs. Bar graph: quantification of wound reduction after 24 h of treatment with 100 µg/ml ox-LDL, compared to the control cells, which were set as 100%, was performed. The results shown in the bar graphs are mean ± SD obtained in three independent experiments. (**A**, right panels) Phase contrast micrographs: representative images of three independent experiments. (B, left panels) Representative fluorescence microscopy micrographs of control and treated female and male cells stained with Phalloidin-FITC (green) and counterstained with Hoechst (blue) Samples were observed with a 63x objective. (**B**, right panel) Quantification of stress fibers were conducted using ImageJ/FIJI software. The corrected total cell fluorescence (CTCF) was quantified. Measurements were performed on 16 cells per condition from three independent experiments. The bars represent minimum and maximal values including all points, and each black dot represents different data points. This analysis revealed a decrease in actin organization in HUVECs treated with ox-LDL compared to controls, as well as a significant decrease in ox-LDL MHUVECs. (**C-D**) Semiquantitative flow cytometric analysis of the ICAM-1 and VCAM-1 levels. The results shown in the bar graphs are mean of the median fluorescence intensity values ± SD obtained in four independent experiments. Dot plots showing a representative experiment of ICAM-1 (**C**, right panels) expression and a representative experiment of VCAM-1 expression (**D**, right panels**)**. See Table 2 for statistical information obtained by the two-way ANOVA test

The ability of HUVECs to migrate towards a damaged site plays an important role in their regenerative response. In this regard, 24 h after scratching confluent HUVECs cultures, it was possible to observe that the migratory capacity of cells treated with ox-LDL was significantly (*p* < 0.001) reduced in both male and female HUVECs (Fig. 1A, left). However, FHUVECs exhibited significantly greater movement capacity than MHUVECs (*p* < 0.001). Interestingly, the cell-free area displayed also significant (*p* < 0.001) treatment and sex (T×S) interaction effects (see Table 2). Data were collected by photographing the lesions at times 0 and 24 h following lesion induction (Fig. 1A, right panel).

**Table 2:** Results of two-way ANOVA

Parameter		CTR		ox-LDL		Significance		
	units	Male	Female	Male	Female	T	S	TxS
Cell free area	µm^2^	100.00 ± 0.00 a	100.00 ± 0.00 a	47.50 ± 2.08 c	65.50 ± 4.20 b	***	***	***
Actin	CTCF x 10^6^	61.75 ± 5.17 a	53.16 ± 4.21 a	47.68 ± 3.53 c	35.78 ± 2.91 d	***	n.s.	***
ICAM-1	a.u.	806.25 ± 106.57	715.50 ± 77.95	1062.50 ± 44.43	874.25 ± 118.22	***	*	n.s.
VCAM-1	a.u.	13.25 ± 1.26	14.28 ± 1.52	16.75 ± 1.71	17.00 ± 1.83	**	n.s.	n.s.
OPA1/GAPDH	a.u.	2.50 ± 0.16 c	3.00 ± 0.22 b	2.00 ± 0.22 d	3.98 ± 0.25 a	*	***	***
MFN2/GAPDH	a.u.	2.05 ± 0.26 ab	1.73 ± 0.13 b	1.18 ± 0.34 c	2.48 ± 0.17 a	n.s.	**	***
DRP1/GAPDH	a.u.	1.00 ± 0.24 b	0.70 ± 0.14 bc	2.55 ± 0.31 a	0.50 ± 0.18 c	***	***	***
Fis1/GAPDH	a.u.	0.50 ± 0.08	1.41 ± 0.17	1.02 ± 0.28	1.62 ± 0.17	**	***	n.s.
PINK/GAPDH	a.u.	2.48 ± 0.34 b	0.50 ± 0.16 c	3.38 ± 0.43 a	0.72 ± 0.10 c	**	***	*
Mitochondrial footprint	µm^2^	3327.6 ± 472.01 b	4957.3 ± 486.5 a	2391.1 ± 393.34 c	4511.7 ± 527.9 a	***	***	*
Branch length	µm	0.28 ± 0.02	0.29 ± 0.02	0.22 ± 0.02	0.23 ± 0.02	***	n.s.	n.s.
Network branches	n	4.42 ± 0.69	2.91 ± 0.49	3.32 ± 0.62	2.18 ± 0.45	***	***	n.s.
H2O2	a.u.	94.00 ± 2.94	87.00 ± 9.56	101.50 ± 2.38	102.25 ± 6.90	**	n.s.	n.s.
O2	a.u.	52.50 ± 5.45 b	75.00 ± 5.89 ab	101.50 ± 31.26 a	71.50 ± 7.33 ab	*	n.s.	**
mROS	a.u.	39.75 ± 2.22 b	42.50 ± 2.38 b	42.25 ± 0.96 b	52.00 ± 2.94 a	***	***	**
CAT/Actin	a.u.	2.00 ± 0.29	2.05 ± 0.21	2.52 ± 0.26	2.45 ± 0.21	**	n.s.	n.s.
SOD1/Actin	a.u.	1.70 ± 0.16	2.95 ± 0.13	2.48 ± 0.17	3.98 ± 0.17	***	***	n.s.
TRX/Actin	a.u.	1.32 ± 0.24	2.05 ± 0.13	0.80 ± 0.08	1.23 ± 0.25	***	***	n.s.
Annexin v	a.u.	3.70 ± 0.22 bc	2.43 ± 0.26 c	11.42 ± 1.16 a	4.10 ± 0.18 b	***	***	***
Bax/Tubulin	a.u.	1.50 ± 0.14 b	1.41 ± 0.10 b	2.45 ± 0.31 a	1.52 ± 0.12 b	***	***	***
Caspase-3/Tubulin	a.u.	0.00 ± 0.00 c	0.00 ± 0.00 c	0.38 ± 0.02 a	0.13 ± 0.05 b	***	***	***
Survivin/Tubulin	a.u.	2.30 ± 0.28 b	2.02 ± 0.13 b	1.12 ± 0.15 c	3.05 ± 0.13 a	n.s.	***	***
LC3II/GAPDH	a.u.	1.18 ± 0.17 c	1.35 ± 0.13 c	2.25 ± 0.26 b	3.15 ± 0.21 a	***	***	**
p62/GAPDH	a.u.	2.23 ± 0.24 b	3.25 ± 0.21 a	3.08 ± 0.34 a	2.90 ± 0.14 a	n.s.	**	***

Data are presented as mean ± standard deviation. HUVECs from male and female donors were either untreated (CTR) or exposed to ox-LDL. T: main effect of treatment; S: main effect of biological sex; T×S: interaction effect between treatment and sex. Statistical significance was determined by two-way ANOVA followed by Tukey’s post-hoc test:

***p < 0.05; **p < 0.01; ***p < 0.001. Different letters within the same row indicate statistically significant differences (*P* ≤ 0.05) between experimental groups and refer to T x S effect. a.u., arbitrary units; CTCF: corrected total cell fluorescence; n: number, n.s.: not significant. Parameters normalized to GAPDH, Actin or Tubulin were assessed via western blot

Since increased cell motility is associated with cytoskeletal remodeling, we assessed whether ox-LDL treatment altered the expression and organization of actin, a cytoskeletal protein involved in maintaining cell shape and motility. For this purpose, both control and ox-LDL treated cells were stained with FITC-phalloidin and observed by a fluorescence microscope. A reduction in stress fibers, especially in male cells was detected (Fig. 1B, left panel). This observation is confirmed by stress fibers quantification. Analysis of the corrected total cell fluorescence (CTCF) revealed a decrease in the organization of actin in HUVECs treated with ox-LDL, compared to the control groups. There was also a significant decrease (*p* < 0.001) in MHUVECs (Fig. 1B, right panel). TxS also displayed significant interaction effects here (*p* < 0.001). This data confirmed the one obtained by scratch test (Fig. 1A).

Moreover, given the observed reduced motility, we next evaluated the effect of ox-LDL on the adhesion molecules ICAM-1 and VCAM-1(Fig. 1C and D, respectively). An upregulation of these proteins was detected by flow cytometry in both male and female cells 24 h after ox-LDL treatment. In particular, we observed a significant increases in ICAM-1 (*p* < 0.001) and VCAM-1 (*p* < 0.01), driven by treatment (see Table 2). However, only ICAM-1 displayed significant (*p* < 0.05) sex-dependent differences at baseline.

### The impact of ox-LDL on mitochondrial dynamics in HUVECs varies by sex

**Fig. 2:**
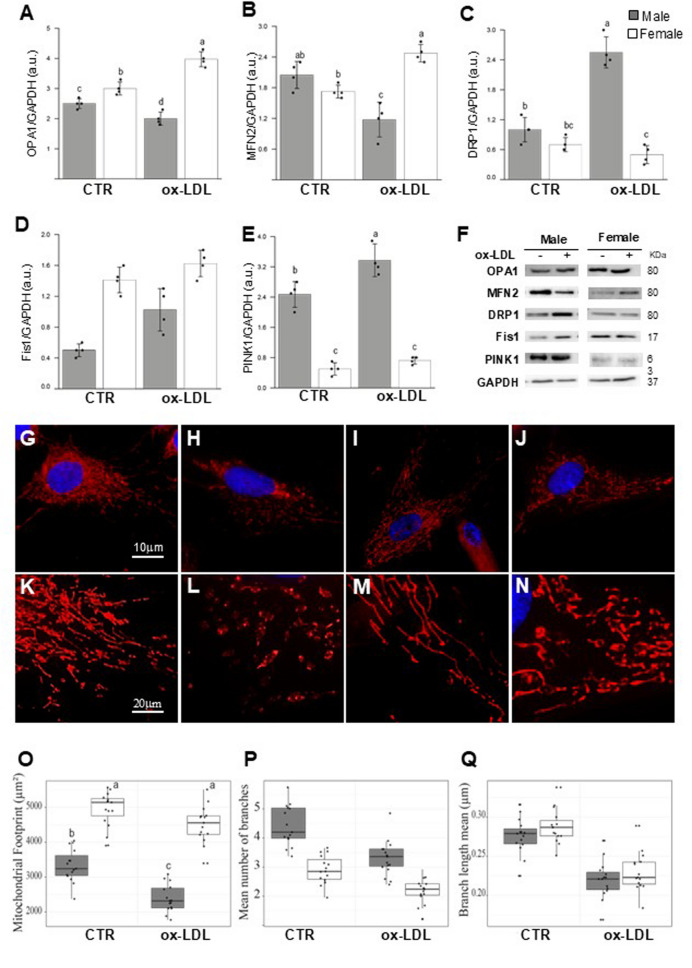
Ox-LDLs affect mitochondrial dynamics in HUVECs. Western blot analyses were performed on proteins such as OPA1 (**A**), MFN2 (**B**), DRP1 (**C**), Fis1 (**D**) and PINK1 (E) involved in mitochondrial dynamics. Bar graphs show densitometry analyses of each protein normalized to GAPDH. Data are expressed as mean ± SD at least of three independent experiments. (**F**) Representative Western blot analysis of the mentioned proteins and GAPDH, which was used as a loading control. (**G-J**) Representative fluorescence microscopy images of control and treated males (**G-H**) and control and treated female cells (**I-J**), stained with Mitotracker Red (red) to highlight the mitochondria and with Hoechst (blue) to highlight the nuclei. Samples were observed with a 63x objective. (**K-N**) The images above have been magnified in select areas. Different mitochondrial morphological parameters were determined using MiNA in Fiji/ImageJ, normalized, and analyzed in comparison to the untreated cell lines to quantify: the mitochondrial footprint (**O**), mean branch length (**P**) and the mean branch number per mitochondrion (**Q**). All data are representative of four analyzed images obtained from four independent dishes from three independent experiments. The bars represent minimum and maximal values including all points, and each black dot represents different data points. See Table 2 for statistical information obtained by the two-way ANOVA test

Under physiological conditions, mitochondria undergo continuous fusion and fission processes, which are necessary for cell survival. To investigate the potential interference of ox-LDL with these processes, the levels of OPA1 and MNF2 (fusion) and DRP1 and Fis1 (fission) were evaluated using Western blotting analysis. Significant differences in OPA1 (*p* < 0.001), MFN2 (*p* < 0.01), PINK1 (*p* < 0.001), DRP1 (*p* < 0.001), and Fis1 (*p* < 0.001) levels were detected between sexes in both controls and ox-LDL treated cells. Ox-LDL triggered extensive changes in mitochondrial dynamics and significantly modulated the expression of MFN2, OPA1 and DRP1. However, Fis1 showed no significant T×S interaction (see Table 2). PINK1 levels were significantly affected by both sex (*p* < 0.001) and treatment (*p* < 0.01), as well as by the T×S interaction (*p* < 0.05) (see Table 2). Moreover, by fluorescence microscopy using Mitotracker Red dye, mitochondrial structure was observed. In Fig. 2G-J representative images of mitochondria in both control and ox-LDL treated cells are shown. Figure 2G–J shows representative images of mitochondria in control and ox-LDL-treated cells in male (Figs. 2G–H) and female (Figs. 2I–J) HUVECs. The images below are a magnification of selected areas (Figs. 2K–N). To investigate the effects of ox-LDL on mitochondrial morphology, analyses of mitochondrial footprint (Fig. 2O), mean branch length (Fig. 2P) and mean branch number per mitochondrion (Fig. 2Q) were performed. Analysis of the total mitochondrial footprint (area) revealed significant main effects of both sex (*p* < 0.001) and treatment (*p* < 0.001), as well as a significant interaction between sex and treatment (*p* = 0.049). Under control conditions, FHUVECs xhibited substantially larger mitochondrial footprints than MHUVECs (4957.3 ± 486.5 μm² vs. 3327.6 ± 472.0 μm²). Importantly, ox-LDL treatment induced a significant reduction in mitochondrial footprint in MHUVECs (*p* < 0.001), whereas FHUVECs showed only a modest, non-significant reduction (Fig. 2O). There was no significant effect of sex on mitochondrial branch length, but a highly significant treatment effect was demonstrated (*p* < 0.001), with no T×S interaction. Ox-LDL treatment reduced the mean branch length by around 20% in both sexes (in males, from 0.276 ± 0.022 μm to 0.220 ± 0.023 μm; in females, from 0.288 ± 0.021 μm to 0.230 ± 0.024 μm), suggesting a conserved mechanism of mitochondrial fragmentation that is independent of biological sex (Fig. 2P).

The number of mitochondrial network branches exhibited significant main effects of both sex and treatment (*p* < 0.001), with no significant interaction. MHUVECs displayed significantly greater network branching at baseline than FHUVEC (4.42 ± 0.67 vs. 2.91 ± 0.49). Ox-LDL treatment induced proportionally similar reductions in network branches in both sexes (−24.9% in males and.

−25.1% in females), suggesting that, although baseline mitochondrial network complexity differs between the sexes, the ox-LDL-induced loss of network connectivity proceeds through a sex-independent mechanism (Fig. 2Q) (see Table 2).

### Ox-LDL alter redox balance in both male and female HUVECs

**Fig. 3:**
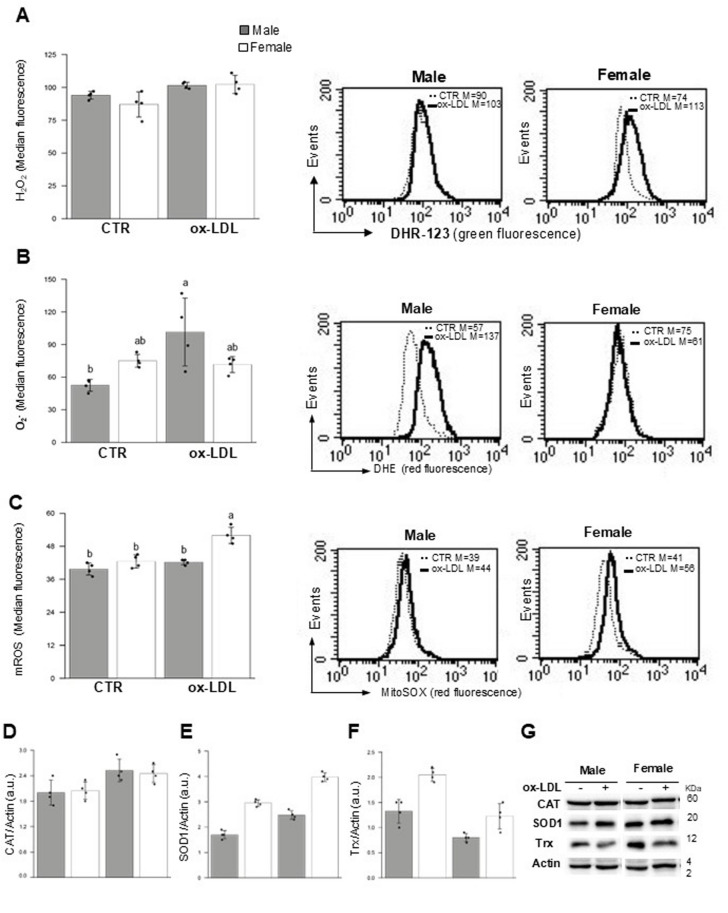
Ox-LDLs alter the redox balance of HUVECs. Semiquantitative flow cytometric analysis of the redox state in both control and ox-LDL treated males and females HUVECs. These analyses were assessed using the cell-permeable fluorophores DHR123 for the detection of H₂O₂ (**A**), DHE for the detection of O₂⁻ (**B**), and MitoSOX for the detection of mROS (**C**). The bar graphs show the mean median fluorescence intensity values ± SD obtained in a least three independent experiments. (**A**, **B**, **C**, right panels) dot plots showing representative experiment of ROS detection. Western blot analyses of the antioxidant enzymes CAT (**D)**, SOD1 (**E)** and Trx (**F**) are shown. Bar graphs show the densitometry analysis of these proteins normalized to Actin. Data are expressed as the mean ± SD of three independent experiments. (**G**) A representative Western blot analysis of the aforementioned proteins and actin, which was used as a loading control, is shown. See Table 2 for statistical information obtained by the two-way ANOVA test. CAT: catalase, SOD1: superoxide dismutase; Trx: thioredoxin

To investigate the effects of ox-LDL on intracellular redox balance, hydrogen peroxide (H_2_O_2_), superoxide anion (O_2_^−^) and mitochondrial ROS (mROS) levels were measured by flow cytometry in both male and female HUVECs before and after 24 h of ox-LDL treatment. The ox-LDL treatment significantly altered the cellular redox state, increasing H₂O₂ (*p* < 0.01) (Fig. 3A), O₂⁻ (*p* < 0.05) (Figure. 3B) and mROS (*p* < 0.001) (Figure. 3 C) levels. Sex-dependent effects were only observed for mROS (*p* < 0.001). Interestingly, T×S interactions emerged for O₂⁻ (*p* < 0.01) and mROS (*p* < 0.01).

At the same time, western blot analyses were conducted on antioxidant enzymes such as catalase (CAT), superoxide dismutase (SOD1) and thioredoxin (Trx). The results showed that ox-LDL treatment markedly altered the antioxidant defences, increasing CAT (*p* < 0.01) (Fig. 3D) and SOD1 (*p* < 0.001) (Fig. 3E) levels and decreasing Trx (*p* < 0.001) (Fig. 3F) levels. While treatment effects were observed across all antioxidant parameters, sex effects were only significant for SOD1 (*p* < 0.001) and Trx (*p* < 0.001). No T×S interactions emerged (see Table 2).

### Sex differences in mitochondrial membrane potential and apoptosis after ox LDL treatment

Given their central role in ROS production and cell death, the effects of ox-LDL on mitochondrial function were assessed in male and female HUVECs, focusing on mitochondrial membrane (MM) hyperpolarization and depolarization by flow cytometry.

**Fig. 4:**
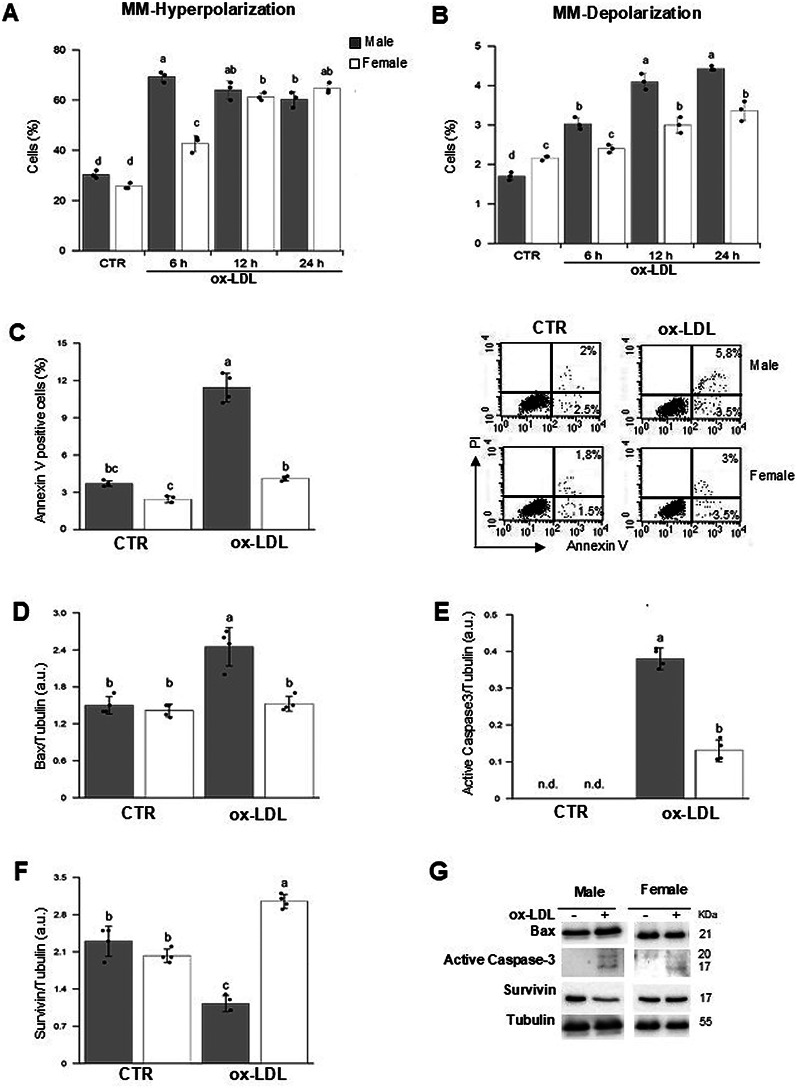
Ox-LDL treatment alters MMP and induces apoptosis in HUVECs. Biparametric flow cytometry was used to analyze mitochondrial membrane (MM) potential in control and ox-LDL-treated HUVECs. The state of hyperpolarization (**A**) and depolarization (**B**) was assessed using the fluorophore JC-1. Bar graphs show the mean ± SD fluorescence intensity values obtained in three independent experiments. (**C**, left panel) Biparametric flow cytometric analysis of apoptosis in male and female HUVECs was performed using Annexin V/propidium iodide. Bar graph represent the mean ± SD of three different experiments. (**C**, right panels) Dot plots showing the percentage of Annexin V-positive cells from representative experiments. (**D–F**) Bar graphs show densitometric analyses of Bax (**D**), active Caspase-3 (**E**), and Survivin (**F**) proteins normalized to Tubulin. Data are expressed as the mean ± SD of four independent experiments. (**G**) Representative Western blot analysis of the above-mentioned proteins and tubulin, used as a loading control. See Tables 2 and 3 for statistical information obtained by the two-way ANOVA test

As shown in Fig. 4A and B sex differences in MM polarization state were found after ox-LDL treatment. In MHUVECs MM hyperpolarization peaked 6 h after ox-LDL treatment, decreasing slowly thereafter. Conversely, in FHUVECs MM hyperpolarization started 6 h after ox-LDL treatment and peaked after 24 h. The results showed that ox-LDL treatment significantly increased the percentage of cells with MM hyperpolarisation (*p* < 0.001) (Fig. 4A). At the same time, sex-dependent effects and T×S interactions (both *p* < 0.001) were observed for MM hyperpolarization (see Table 2).

**Table 3:** Results of two-way ANOVA

Treatment	Sex	MM-Iperpolarisation	MM-Depolarisation
CTR	Male	30.33 ± 1.53 d	1.70 ± 0.10 d
	Female	25.67 ± 1.15 d	2.17 ± 0.06 c
6 h ox-LDL	Male	69.33 ± 2.08 a	3.03 ± 0.15 b
	Female	42.67 ± 3.21 c	2.40 ± 0.10 c
12 h ox-LDL	Male	64.00 ± 3.61 ab	4.10 ± 0.20 a
	Female	61.33 ± 1.53 b	3.00 ± 0.20 b
24 h ox-LDL	Male	60.33 ± 3.06 b	4.43 ± 0.06 a
	Female	64.67 ± 2.08 ab	3.37 ± 0.25 b
Significance	T	***	***
	S	***	***
	TxS	***	***

Data are presented as mean ± standard deviation. HUVECs from male and female donors were either untreated (CTR) or exposed to ox-LDL. T: main effect of treatment; S: main effect of biological sex; T×S: interaction effect between treatment and sex. Statistical significance was determined by two-way ANOVA followed by Tukey’s post-hoc test: ***p < 0.001. Different letters within the same row indicate statistically significant differences (*P* ≤ 0.05) between experimental groups and refer to T x S effect

As shown in Fig. 4B, the percentage of cells exhibiting MM depolarisation levels was also significantly impacted by treatment (*p* < 0.001), sex (*p* < 0.001) and the T×S interaction (*p* < 0.001), as shown in Table 2.

It is known that an excess of ROS production and MM depolarization can lead to the activation of programmed cell death or apoptosis. Using a flow cytometry Annexin V kit, the percentage of apoptotic cells was measured at basal levels and after ox-LDL treatment. As shown in Fig. 4C, a significant difference (*p* < 0.001) in the percentage of apoptotic cells was observed following ox-LDL treatment. This difference was also significantly impacted by sex (*p* < 0.001) and the T × S interaction (*p* < 0.001), as can be seen in Table 2. Western blot analysis was used to assess apoptotic markers, including Bax and active Caspase-3. As shown in Fig. 4D and E, densitometric analysis revealed that treatment, sex and the T×S interaction significantly altered the expression of these proteins (all *p* < 0.001; see Table 2). To investigate whether the low mortality detected in FHUVECs after ox-LDL treatment was promoted by the action of inhibitors of apoptosis proteins (IAPs), we also measured the content of Survivin, the smallest member of the IAP gene family. Interestingly, densitometric analysis of Survivin (Fig. 4F) revealed that the levels of this protein were significantly altered by sex and the T×S interaction (all *p* < 0.001) (see Table 2). Figure 4G shows representative western blotting images of all the proteins.

### Treatment of ox-LDL shows sex differences in autophagy process

**Fig. 5:**
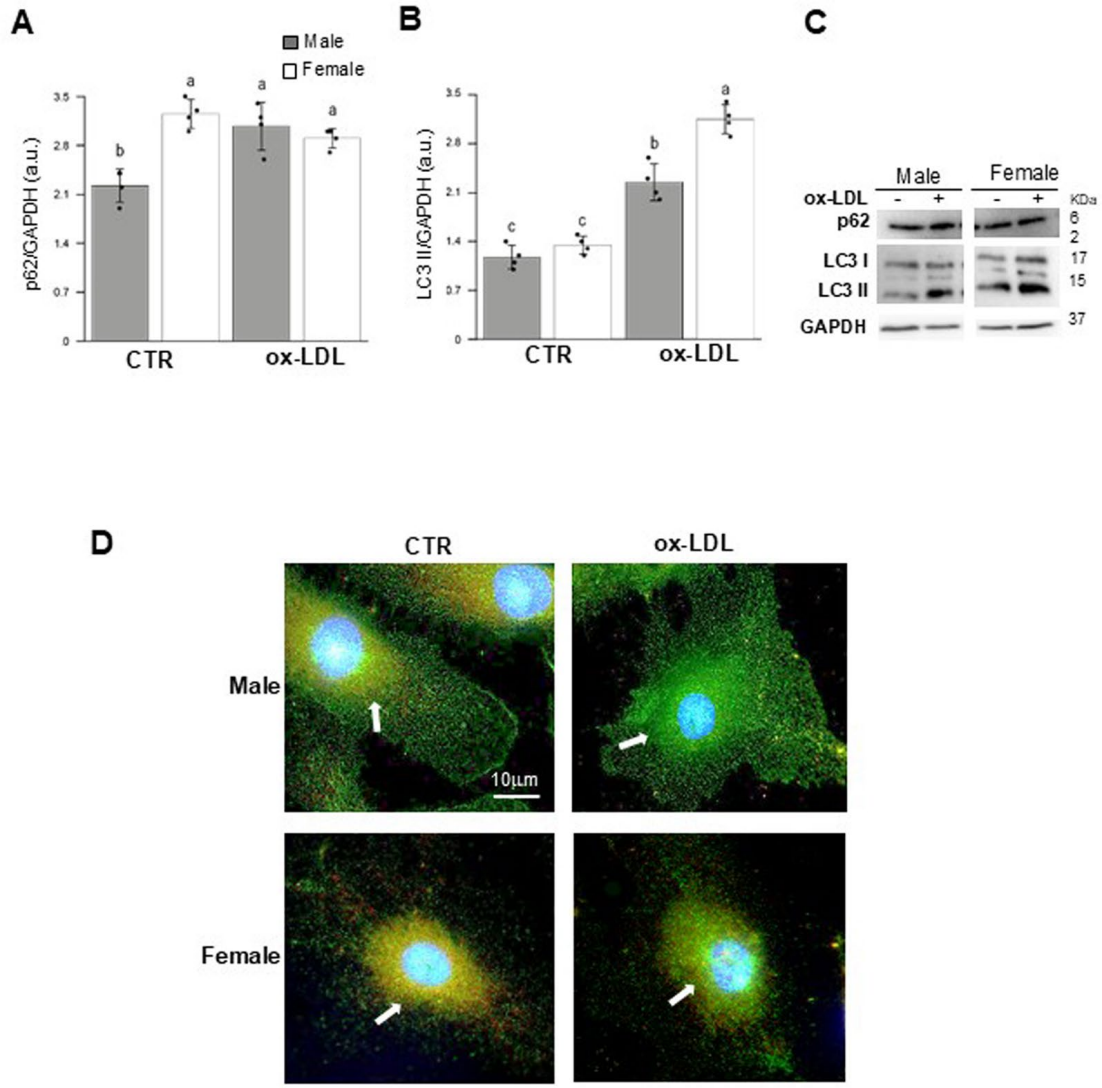
Ox-LDLs induce autophagy in HUVECs. Western blot analyses of p62 and LC3 proteins involved in autophagy. (**A-B**) Bar graphs show densitometry analyses of p62 and LC3 proteins normalized to GAPDH. Data are expressed as mean ± SD of a least three independent experiments. Representative Western blot analysis of the aforementioned proteins and GAPDH, which was used as a loading control. (**C**) Representative fluorescence microscopy micrographs of control and treated cells stained with LC3 (green) and p62 (red) to visualize autophagic vesicles and counterstained with Hoechst (blue) to visualize the nuclei. Samples were observed with a 63x objective. Note the decrease in yellow areas (white arrow) indicating the colocalization of LC3 and p62 in MHUVECs. See Table 2 for statistical information obtained by the two-way ANOVA test

Literature data report that autophagy is gradually impaired during the development of atherosclerosis. To this end, the autophagy proteins p62 and LC3 were assessed using Western blotting in both control and ox-LDL-treated cells (Fig. 5). As shown in Fig. 5A, p62 content was significantly affected by treatment (*p* < 0.001), sex (*p* < 0.001), and the T×S interaction (*p* < 0.01), as detailed in Table 2. Conversely, LC3 levels were significantly affected only by sex (*p* < 0.01) and the T×S interaction (*p* < 0.001) (see Table 2 for details). Figure 5C shows representative western blotting images of p62 and LC3 protein. In Fig. 5D representative fluorescence microscopy images showing p62 (red) and LC3 (green) distribution are depicted. In the images, after ox-LDL treatment, autophagic vacuoles (yellow area) and a reduced colocalization of p62 with LC3 in MHUVECs were evident. These data are consistent with the results obtained by Western blotting.

## Discussion

Endothelial dysfunction can promote atherosclerosis and increase morbidity and mortality due to major cardiovascular events. It is recognized that cardiovascular risk can also occur in the fetus or newborn exposed to maternal nutritional excess [41]. Furthermore, in utero exposure to ox-LDL is reported to be associated with early vascular remodelling and pro-atherogenic changes in the fetal endothelium, potentially predisposing the offspring to long-term cardiovascular risk [42–44].

In recent years, treatment of HUVECs with ox-LDLs has been frequently used as a model to study the pathogenesis of atherosclerosis [45, 46]. There are still many gaps in our understanding of the influence of sex on atherosclerosis mechanisms, and many, if not all, studies do not report cell sex.

In our study, we analyzed the response of HUVECs from male and female newborns to the damage induced by ox-LDL treatment.

Literature data show that ox-LDL induce smooth muscle cells proliferation and migration and it is an important pathogenic factor that induces excessive oxidative stress and apoptosis in vascular ECs [47]. In the present study, by using scratch test, we found that ox-LDL exposure reduced cell motility in both male and female cells. The data are also supported by the reduction in stress fibres detected through immunofluorescence microscopy analysis and CTCF. Both methods revealed a decrease in the organization of the actin cytoskeleton in HUVECs treated with ox-LDL, with a more significant decrease detected in MHUVECs. Interestingly, we also found that FHUVECs treated with ox-LDL exhibited significantly better motility and thus repair capacity than male cells. These findings are also supported by the expression of certain adhesion molecules, such as ICAM-1 and VCAM-1, which are significantly upregulated only in male cells after ox-LDL treatment. It is known that overexpression of adhesion molecules by ECs recruits inflammatory monocytes/leukocytes into the vascular wall and initiates the atherosclerotic process [48, 49].

Moreover, we found that ox-LDL treatment increased the levels of molecules involved in mitochondrial fusion in female cells and increased the levels of molecules involved in mitochondrial fission in male cells. These results are consistent with existing literature, which suggests that ox-LDL induce increased fission and decreased fusion [50,51]. Indeed, it has previously been reported that ox-LDL treatment can induce endothelial apoptosis associated with DRP1-related mitochondrial fission [50, 51]. Furthermore, a high content of PINK1 was observed in both untreated and treated MHUVECs. PINK1 is a serine/threonine protein kinase that is known to regulate mitochondrial dynamics. In healthy mitochondria, PINK1 is maintained at low levels due to post-transcriptional regulation and rapid degradation by proteolysis. In damaged mitochondria PINK1 degradation is inhibited, leading to PINK1 accumulation within the damaged mitochondria [52]. This evidence further supports our conclusion that ox-LDL treatment induces programmed cell death or apoptosis in male cells.

The consistent reduction in mitochondrial branch length (approximately 20%) observed in both male and FHUVECs following ox-LDL treatment is consistent with the established paradigm of oxidative stress-induced mitochondrial fission. Ishida et al. (2024) documented that excessive mitochondrial fission, driven primarily by DRP1 activation, contributes to vascular pathologies via GTP-dependent constriction and fragmentation of mitochondria [53]. Similarly, Cueva-Vargas et al. [54] recently demonstrated that DRP1 phosphorylation at serine 616 promotes its translocation to the mitochondrial outer membrane, thereby triggering mitochondrial fission and ROS production. The reduction in branch length observed here is consistent with this DRP1-mediated fission mechanism, as shorter mitochondrial segments are a hallmark of fragmented mitochondrial networks. The proportional reduction in network branches (by ~ 25%) in both sexes provides further support for a conserved mechanism of mitochondrial network disruption. This loss of network connectivity likely reflects the physical separation of previously connected mitochondria due to excessive fission, as well as the inability of damaged mitochondria to undergo compensatory fusion [12]. Our findings complement those of Liang et al. (2021), who reported that ox-LDL significantly reduce mitochondrial membrane potential and impair mitochondrial function in HUVECs [55]. This establishes mitochondrial dysfunction as a pivotal feature of ox-LDL-induced endothelial pathology.

The most striking finding of our study was the significant sex-by-treatment interaction for mitochondrial footprint. FHUVECs exhibited remarkable resistance to ox-LDL-induced reduction in mitochondrial area (9% vs. 28% in males). This sex-dependent protection is strongly supported by recent mechanistic studies. For example, Liu et al. (2019) demonstrated that the cardiovascular protection observed in females is directly linked to estrogen-mediated reduction in ox-LDL responsiveness. They showed that estrogens antagonize ox-LDL-stimulated MMP12 secretion and reduce atherosclerotic burden [56].

The mitochondrial-level protection observed is consistent with recent evidence demonstrating that female mitochondria possess greater intrinsic resistance to oxidative stress. Studies on cardiac tissue have shown that mitochondria from female cardiomyocytes are more resistant to acute stress than those from males. Specifically, 17β-estradiol treatment protects against mitochondrial membrane potential collapse induced by TNF-α [57]. The preservation of the mitochondrial footprint in female-derived cells despite exposure to ox-LDL suggests that there may be sex-dependent protective mechanisms that target mitochondrial mass homeostasis specifically. Although both sexes experience mitochondrial fragmentation, as evidenced by reduced branch length, female cells appear to maintain total mitochondrial mass through enhanced biogenesis or reduced mitophagy. This could be a vital adaptive mechanism that preserves cellular bioenergetic capacity in the face of oxidative stress.

Our findings demonstrate that ox-LDL induce substantial morphological alterations to the mitochondria of HUVECs, with striking sex-dependent differences in the magnitude of the response to certain parameters. These results shed new light on the sex-dependent nature of endothelial mitochondrial damage and are important for understanding sex differences in atherosclerotic cardiovascular disease.

ROS are reactive oxygen-containing chemical species that are essential for cell signaling and homeostasis in a biological condition. However, in cases of environmental disorders (e.g. oxidative stress), a dramatic increase in ROS production may be observed, which could potentially result in significant damage to cell structures [58]. Considering that ROS act as key mediators in ox-LDL-induced cellular responses, in this study we measured H_2_O_2_, O_2_^−^ and mROS as well as some antioxidant enzymes, such as CAT, SOD1 and Trx. Ox-LDL treatment markedly altered the cellular redox state, increasing ROS levels and modulating antioxidant defences. Treatment effects were observed across all redox parameters, while sex effects were significant for CAT, SOD1, Trx and mROS. T×S interactions also emerged for O₂⁻ and mROS, indicating the coexistence of shared oxidative stress responses and sex-dependent redox adaptations to ox-LDL exposure. Interestingly, the higher rate of death in MHUVECs could be explained by the increased O_2_^−^ levels associated with a smaller increase in SOD1 levels. Conversely, the increased mROS levels in FHUVECs could be counteracted by high Trx levels. Our data demonstrate that, following exposure to ox-LDL, male-derived HUVECs exhibit significantly higher susceptibility to apoptosis than FHUVECs. This sex-dependent vulnerability coincides with a substantial increase in O_2_^−^ production and a notable decrease in Trx levels in male cells. These observations are consistent with the well-established role of ox-LDL in promoting oxidative stress and ECs death [59]. The existence of sex differences in redox homeostasis and oxidative stress responses is increasingly recognised as biologically meaningful. In multiple cell types and in vivo models, males tend to exhibit higher ROS accumulation and weaker antioxidant defences than females. This may be due to the differential regulation of antioxidant pathways and the effects of sex hormones on redox enzymes [60]. Specifically, MHUVECs have been reported to produce more ROS in response to inflammatory stimuli than female cells, suggesting an inherently pro-oxidative ECs phenotype in males [33]. The reduction in Trx observed in MHUVECs provides a mechanistic clue: Trx is a central thiol-based antioxidant system that mitigates oxidative damage and regulates apoptotic signalling. A loss of Trx can impair a cell’s ability to scavenge ROS and maintain redox balance, thereby sensitising cells to apoptosis induced by oxidative stress. While there are few direct reports on sex differences in Trx in HUVECs, studies in other contexts demonstrate that imbalances in ROS production and antioxidant defences are associated with LDL oxidation and endothelial dysfunction [61]. Our findings are consistent with the idea that female cells often have stronger antioxidant defences, which may be due to estrogen-driven expression of redox regulators or differences in NADPH oxidase activity [62]. These factors reduce net oxidative burden and apoptotic susceptibility. This could explain why FHUVECs maintain lower O_2_^−^ levels and better preserve Trx under the same ox-LDL challenge, dampening the apoptotic cascade compared to male cells.

It is known that excess of ROS production can lead to alteration of mitochondrial membrane potential and ultimately to apoptosis [62]. In this study, an increase in cells with MM hyperpolarization was found in both male and female cells after ox-LDL treatment. However, while the maximum peak of cells with MM hyperpolarization was measured 6 h after ox-LDL treatment in males, it was detected after 24 h of treatment in females. These data highlight that, after treatment with ox-LDL, ROS production in males occurs earlier than in females. Furthermore, we found that, following ox-LDL treatment, the percentage of cells with MM depolarisation increased in a time-dependent manner in both males and females, with a more significant increase observed in male cells.

It is important to note that the loss of MM was accompanied by a significant increase in the percentage of apoptotic cells only in males.

ROS production can induce apoptosis through the mitochondrial activation of Caspase-3, a crucial executioner protease in the apoptotic pathway [63]. In our study, we found that after ox-LDL treatment the content of Bax and Caspase-3 activity significantly increased in male cells, indicating a significant sex difference.

Remarkably, after ox-LDL treatment female cells showed high levels of Survivin, an IAP protein that inhibits caspase activation and negatively regulates apoptosis [64].

Oxidative stress can also activate an alternative pathway to apoptosis known as autophagy, a catabolic process that recycles damaged organelles and cellular components to maintain intracellular homeostasis [65], thereby playing an important role in preventing atherosclerosis. However, autophagy is gradually impaired as atherosclerosis develops. A growing body of research has revealed a strong link between dysfunctional autophagy and atherosclerosis.

p62 plays a crucial role in the autophagy process. Elevated levels of this protein often indicate autophagy dysfunction [66]. The aggregation of p62 has been observed in atherosclerotic plaques [67], and as the plaque burden and age increase, the level of p62 in the plaques tends to rise [68]. One of the major limitations of this work is the lack of an in-depth study on possible alterations to the autophagic process. Nevertheless, our data indicate alterations to some autophagy marker proteins, including p62 and LC3. Our study demonstrated that 24 h after ox-LDL treatment p62 levels increased only in MHUVECs, whereas LC3II levels increased significantly in both male and female cells. Although a previous study [32] demonstrated no differences in baseline levels of the p62 protein between male and female HUVECs, our contradictory results could be due to the demographic characteristics of the mothers. Here, the mothers are older and weigh less than in the previous study, and it is known that autophagy fits with metabolic profile and age [69]– [70].

Interestingly, although Survivin is often described as being inversely associated with autophagy in certain models, particularly in cancer, our results suggest that exposure to ox-LDL triggers an increase in both autophagic markers and Survivin expression. This coexistence likely reflects a coordinated cytoprotective response in which autophagy mitigates cellular damage and Survivin limits the execution of apoptosis, ultimately contributing to reduced cell death in female cells.

Our data show that male and female cells respond differently to ox-LDL treatment: MHUVECs undergo apoptosis and experience changes in autophagy-associated protein levels, whereas female cells appear unaffected by alterations in autophagy-associated protein levels. In conclusion, our study demonstrates that sex-related differences in mitochondrial function are established during the prenatal period, supporting previous findings obtained with different stimuli [32, 35] and are particularly pronounced in MHUVECs after ox-LDL treatment.

ECs treated with ox-LDL experience increased oxidative stress, which leads to alterations in mitochondrial dynamics, reduced autophagy, and increased cell death. Female cells appear to tolerate oxidative stress better and have a greater repair capacity than male cells. Considering the data obtained, we can conclude that the response of HUVECs to the damaging effects of ox-LDL is influenced by sex.

However, since this is an in vitro study, it has limitations in representing the real vascular environment and the immune and systemic interactions essential for endothelial dysfunction. Indeed, in vivo, ox-LDL are potential promoters of vascular inflammation and, in addition to endothelial cells, stimulate monocytes, macrophages, vascular smooth muscle cells, and other inflammatory cells to produce pro-inflammatory cytokines such as TNF-α and IL-6, and the chemoattractant protein MCP-1, which induces the movement of inflammatory cells into plaques [71, 72].

To counteract ox-LDL-induced oxidative damage, future studies could be conducted using N-acetylcysteine, a potent antioxidant with anti-inflammatory properties, or polyphenol compounds such as resveratrol.

Sex-dependent endothelial responses are relevant to early cardiovascular risk because males and females have biological, genetic, hormonal, and immunological differences that significantly influence endothelial function.

A greater understanding of the impact of sex on the response to cholesterol-induced stress will provide insights into the mechanisms underlying endothelial dysfunction. This will also offer potential biomarkers and/or innovative preventive approaches to combat cardiovascular disease and ensure personalized care.

## Supplementary Information

Supplementary Material 1.

Supplementary Material 2.

## Data Availability

No datasets were generated or analyzed during the current study.
