# Supplementary figures and images for: Sex differences in human umbilical vein endothelial cells following ox-LDL injury
Source: Biol Sex Differ. 2026 Feb 7;17:48. doi: 10.1186/s13293-026-00845-5 (PMC12977849; doi:10.1186/s13293-026-00845-5)

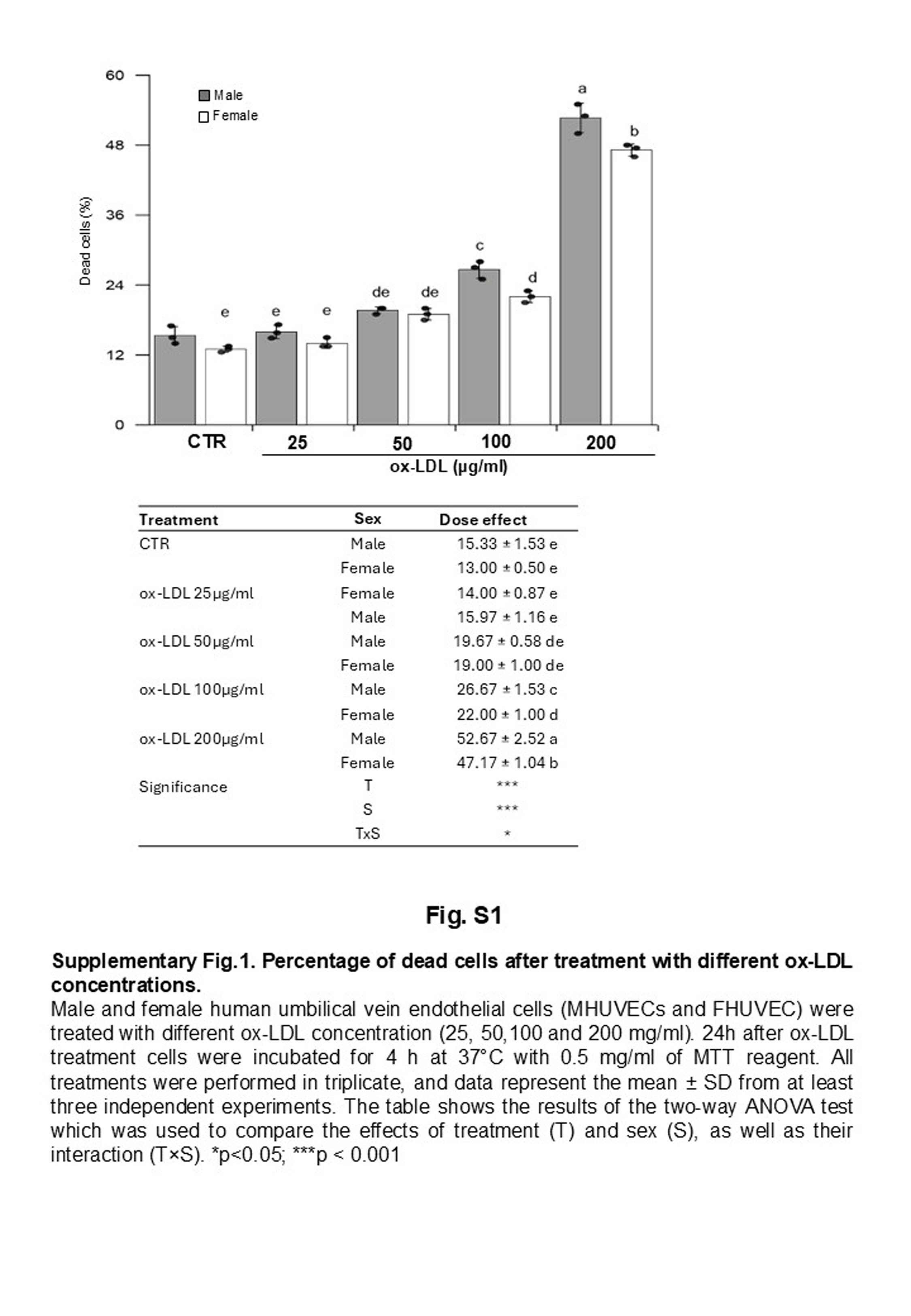

Supplement: Supplementary file 1 — Supplementary Material 1. [file 13293_2026_845_MOESM1_ESM.tif]

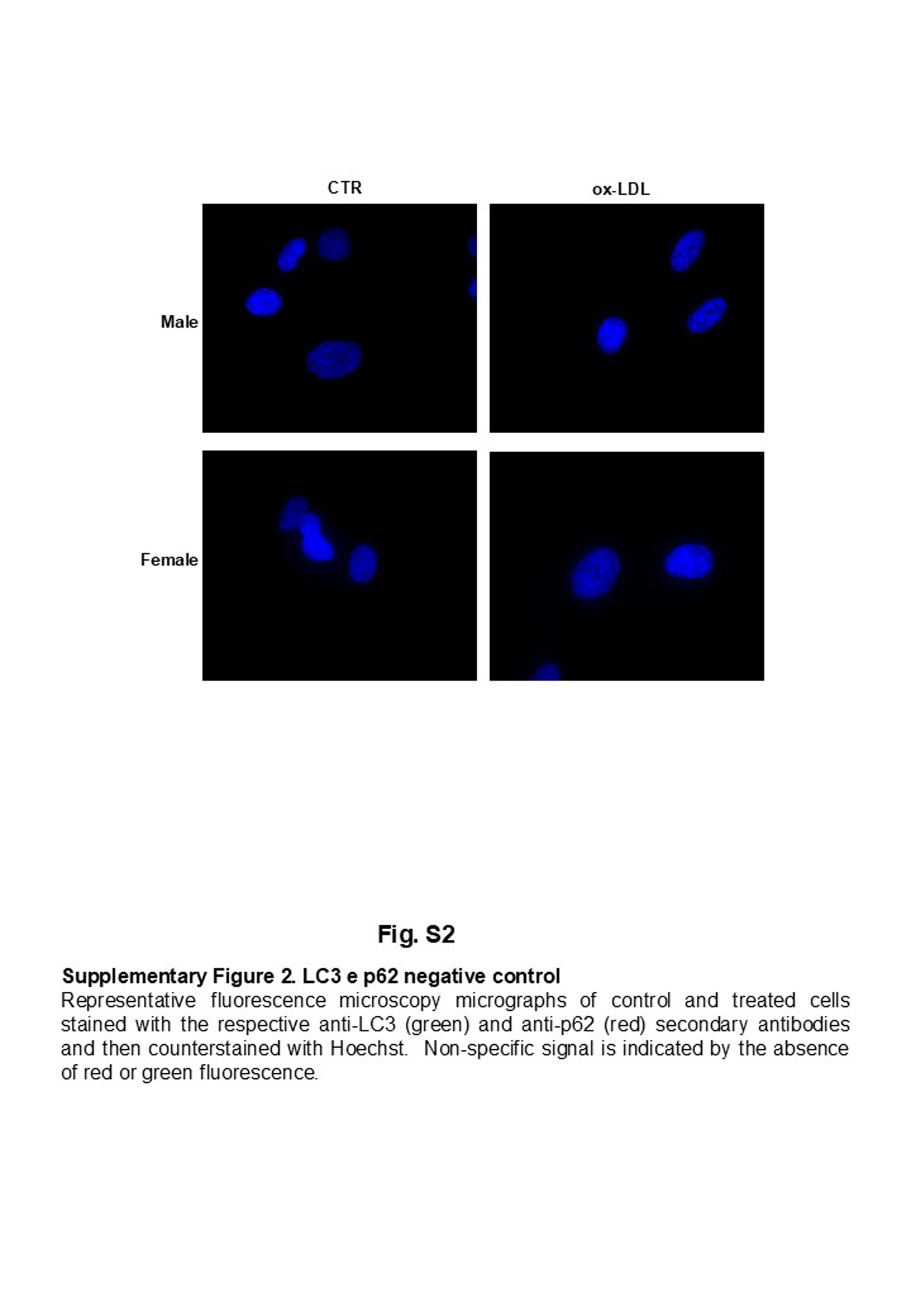

Supplement: Supplementary file 2 — Supplementary Material 2. [file 13293_2026_845_MOESM2_ESM.tif]
